# Amphibian fauna of Pakistan with notes on future prospects of research and conservation

**DOI:** 10.3897/zookeys.1062.66913

**Published:** 2021-10-15

**Authors:** Muhammad Rais, Waseem Ahmed, Anum Sajjad, Ayesha Akram, Muhammad Saeed, Hannan Nasib Hamid, Aamina Abid

**Affiliations:** 1 Herpetology Lab, Department of Wildlife Management, Pir Mehr Ali Shah Arid Agriculture University Rawalpindi, Rawalpindi, 46300, Pakistan Pir Mehr Ali Shah Arid Agriculture University Rawalpindi Pakistan; 2 Department of Environmental Sciences, International Islamic University, Islamabad, Pakistan International Islamic University Islamabad Pakistan

**Keywords:** Black-spined toad, Data Deficient, chytrid, endemism, extinction, inclusive conservation, intrinsic value, South Asia

## Abstract

Research on amphibians and their conservation have gained worldwide attention, as the group includes the highest number of threatened and Data Deficient species when compared to other vertebrates. However, amphibians have long been neglected in wildlife conservation, management decisions, policy making, and research agendas in Pakistan. In this paper, an annotated checklist of the 21 amphibian species of Pakistan, a key to their identification, and detailed discussions on variation in species, including the genera *Minervarya* and *Sphaerotheca*, are provided. We found a statistically significant difference in the morphometric measurements of males but non-significant difference in the females of the two forms (rusty dorsum and dotted dorsum) of *S.maskeyi*. Some genera, such as *Microhyla*, *Uperodon*, *Minervarya*, *Allopaa*, *Chrysopaa*, *Euphlyctis*, *Nanorana*, and *Sphaerotheca*, in Pakistan are in need of additional data for molecular and morphological comparisons with taxa in other South Asian countries. The predicaments of amphibian research in Pakistan are discussed, gaps identified, and suggestions are made. Although the occurrence of chytrid fungus in Pakistan is predicted of low likelihood, a lack of data merits studying the prevalence of the fungus, particularly in the northern regions of the country which exhibit complex and dynamic ecosystems. It is recommended that systematic and coordinated surveys are conducted throughout the country to build a database of species occurrences and distributions. Additionally, the monitoring of wild populations and threat mitigation, as well as appropriate legislation, are suggested as long-term measures. By adopting an inclusive wildlife conservation approach in Pakistan, amphibians could be integrated into wildlife conservation and management efforts.

## Introduction

Amphibians are bioindicators of an ecosystem’s health and may also serve as a biological control of crop and forest pests (Attademo et al. 2005; Kanagavel et al. 2017). Additionally, various important compounds have also been extracted from their skin and eggs for medical applications (Erspamer 1971; Clarke 1997). Amphibians are sometimes kept as pets (Gerson 2012) and are also a source of food (protein) for people in many regions of the world (Marineros 2007). The number of currently described amphibian species is 8378 (Frost 2021).

The First Herpetological Congress, organized in 1989, presented alarming findings about the decline in amphibian populations which was presumed to have started in the early 1970s in the United States, certain Central American countries, and in northeastern Australia (Czechura and Ingram 1990; Drost and Fellers 1996; Burrowes et al. 2004). Amphibians include the highest number of Data Deficient species (>1500 species) (Morais et al. 2013) and the highest percentage (>40 %) of threatened species among all vertebrate groups. Bishop et al. (2012) categorized threats to amphibians into two groups. The first group of threats included habitat destruction and fragmentation, exotic invasive species, and over-exploitation. The second group, which is more poorly understood, includes the threats of infectious diseases and global climate change. Approximately 700 amphibian species are known to have been affected globally by the chytrid fungus, *Batrachochytriumdendrobatidis*. This fungus has extirpated about 90 amphibian species and has caused population declines of over 500 species (Rosenblum et al. 2010; Lips 2016; Scheele et al. 2019).

This paper provides an annotated checklist of the 21 amphibian species of Pakistan and keys to their identification. The predicaments of amphibian research in Pakistan are discussed and knowledge gaps identified. Suggestions are made on how to proceed with research and conservation of amphibians in the country.

## Materials and methods

The available historical as well as recent literature on the amphibians of Pakistan was critically reviewed. We collected data on the morphology of 10 amphibian species (*N* = 158) (Suppl. material 1, Table S1) beginning in 2015 from the areas of Rawalpindi, Islamabad, and Gilgit-Baltistan. We used published literature (Murray 1884; Khan and Tasnim 1989; Auffenberg and Rehman 1997; Dutta 1997; Stöck et al. 1999; Khan 2006; Dufresnes et al. 2019; Ali et al. 2020) on other species in the development of the identification keys.

We studied morphological differentiation of the two forms of *Sphaerothecamaskeyi*: uniform rusty-colored dorsum (*n* = 9, Fig. 3F) and dorsum olive with dotted pattern (n = 29, Fig. 3E). We performed a principal components analysis (PCA) on 23 morphometric measurements separately on males and females (Borzée et al. 2013) in XLSTAT to reduce the studied measurements into fewer significant variables (*r* > 0.90) (see variable 1–23 in Suppl. material 1, Table S2a, S2b). Principal components analysis (PCA) is a variable-reduction technique that shares many similarities to exploratory factor analysis. The aim is to reduce a larger set of variables into a smaller set of “artificial” variables, called “principal components”, which account for most of the variance in the original variables. We then conducted a multivariate generalized linear model (one-way MANOVA) to examine if there were any differences (*a* = 0.05) between categorical predictor variables in the two forms (in males and females separately) on continuous response variables (obtained after PCA with *r* > 0.90) in SPSS 22.

## Results

There are 21 species of amphibians (order Anura) in Pakistan, belonging to four families: Bufonidae Gray, 1825, Megophryidae Bonaparte, 1850, Microhylidae Günther, 1858, and Dicroglossidae Dubois, 1987. The identification keys of amphibian families and species of Pakistan are as follows:

### Key to amphibian families of Pakistan

**Table:** 

1	Parotid glands present	**Bufonidae**
–	Parotid glands absent	**2**
2	Pupil vertical	**3**
–	Pupil horizontal	**Dicroglossidae**
3	Head and mouth narrow, body smooth with few smooth small tubercles	**Microhylidae**
–	Head and mouth broad, body heavily warty, a distinct elevated post orbital ridge	**Megophryidae**

### Key to species

**Bufonidae**

**Table:** 

1	Head with cranial crest	**2**
–	Head without cranial crest	**3**
2	Only supraorbital crest, tympanum indistinct	***Duttaphrynushimalayanus* (Günther, 1864) (Fig. 1C)**
–	Supraorbital, canthal, post orbital, orbitotympanic crest, tympanum distinct ***Duttaphrynusmelanostictus* (Schneider, 1799) (Fig. 2A)**
3	Interorbital space is smaller or nearly equal to the internarial space	**4**
–	Interorbital space a little wider than the upper eyelid	**6**
4	Parotid glands are inconspicuous, subarticular tubercles single under toes; often double on first, second, and, in some, third finger	***Bufotesbaturae* (Stöck et al., 1999) (Fig. 1B)**
–	Parotid glands conspicuous, toes with double subarticular tubercles	**5**
5	Dorsal pattern of longitudinal stripes, three on each side	***Bufoteslatastii* (Boulenger, 1882) (Fig. 1D)**
–	Dorsum gray, with greenish spotting, a dark blotch on the upper eyelid	***Bufotessurdus* (Boulenger, 1891) (Fig. 1A)**
6	Tibial gland absent	**7**
–	Tibial gland present, tarsal fold indicated by weak spinulated line	***Duttaphrynusstomaticus* (Lütken, 1864) (Fig. 2C)**
7	Dorsum uniformly olive, interorbital space slightly concave, parotids depressed	***Duttaphrynusolivaceus* (Blanford, 1874) (Fig. 2B)**
–	Dorsum with green pattern	**8**
8	Dorsum with scattered green spots	***Bufoteszugmayeri* (Eiselt and Schmidtler, 1973) (Fig. 1E)**
–	Dorsum heavily green with occasional light spots, Dorsal tubercles are not so prominent, rather they are flat	***Bufotespseudoraddei* (Mertens, 1971) (Fig. 1F)**

**Figure 1.**
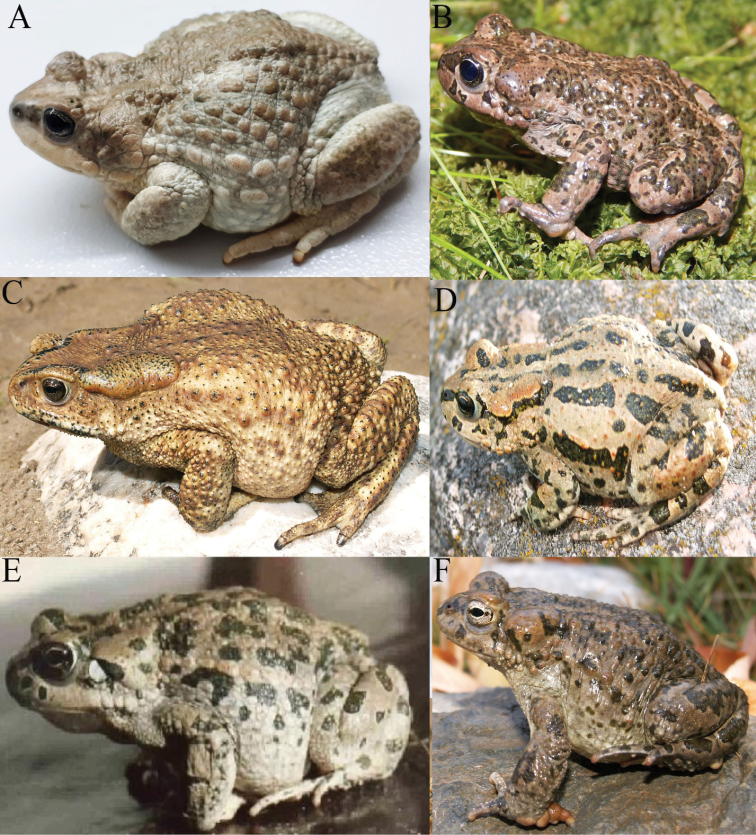
**A** Iranian Toad (*Bufotessurdus*) **B** Batura Toad (*Bufotesbaturae*) **C** Himalayan Toad (*Duttaphrynushimalayanus*) **D** Ladakh Toad (*Bufoteslatastii*) **E** Baloch Green Toad (*Bufoteszugmayeri*) **F** Swat Green Toad (*Bufotespseudoraddei*). Photographers: Dr Spartak Litvinchuk (**A–D**, **F**); Muhammad Sharif Khan (**E**).

**Figure 2.**
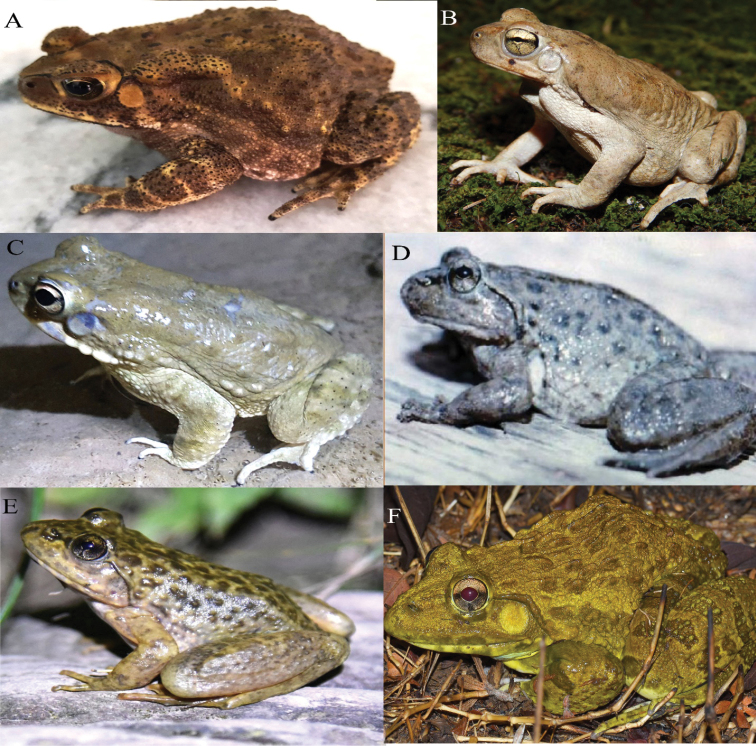
**A** Asian Common Toad or Black-spined Toad (*Duttaphrynusmelanostictus*)**B** Olive Toad (*Duttaphrynusolivaceus*) **C** Indus Valley Toad (*Duttaphrynusstomaticus*) **D** Kashmir Torrent Frog (*Allopaabarmoachensis*) **E** Hazara Torrent Frog (*Allopaahazarensis*) **F** Indus Valley Bull Frog (*Hoplobatrachustigerinus*). Photographers: Dr Muhammad Rais (**A**, **C**, **E**); Dr Spartak Litvinchuk (**B**); Muhammad Sharif Khan (**D**); Janis Czurda (**F**).

**Figure 3.**
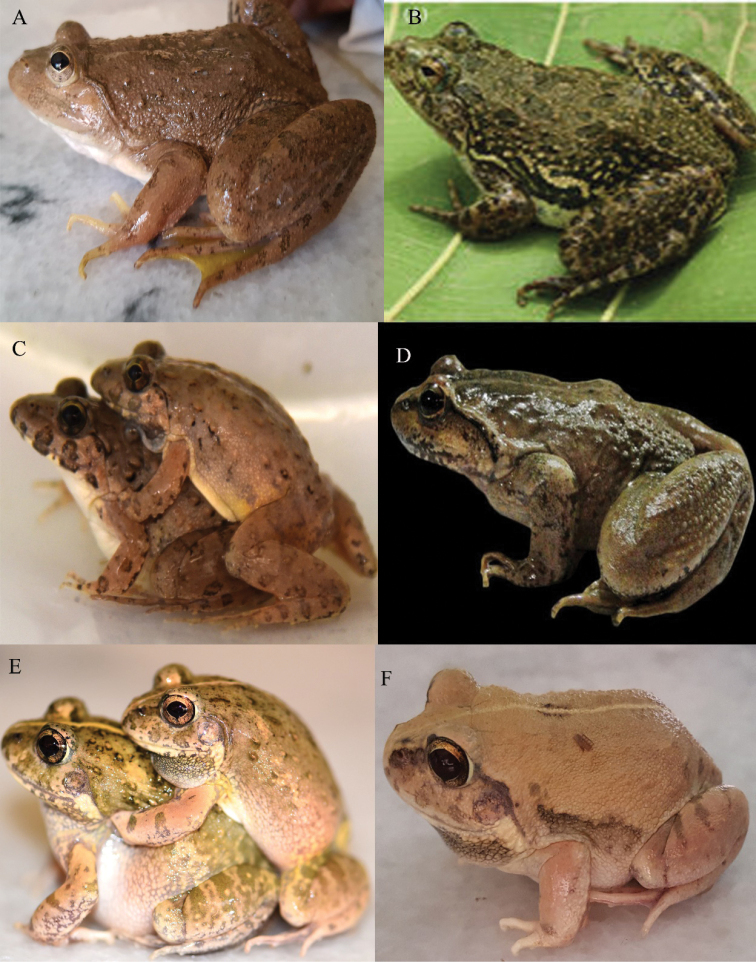
**A** Skittering Frog (*Euphlyctiscyanophlyctis*) **B** Skittering Frog (*Euphlyctiskalasgramensis*) **C** Pierrei’s Cricket Frog (*Minervaryapierrei*) **D** Murree Hills Frog (*Nanoranavicina*) **E**, **F** Burrowing Frog (*Sphaerothecamaskeyi*). Photographers: Dr Muhammad Rais (**A**, **C–F**); Waqas Ali (**B**).

**Megophryidae**

**Table:** 

1	Head and mouth broad, body heavily warty, a distinct elevated post orbital tuberculate ridge, tympanum indistinct	***Scutigeroccidentalis* (Dubois, 1978) (Fig. 4B)**

**Microhylidae**

**Table:** 

1	Tongue elliptical, adult <30 mm, body dorsum with elongated, light brown, large, branched blotch	***Microhylanilphamariensis* (Howlader et al., 2015**a) **(Fig. 4C)**
–	Tongue oval, adult 50–60 mm, dorsum with brown reticulation	***Uperodonsystoma* (Schneider, 1799) (Fig. 4D)**

**Figure 4.**
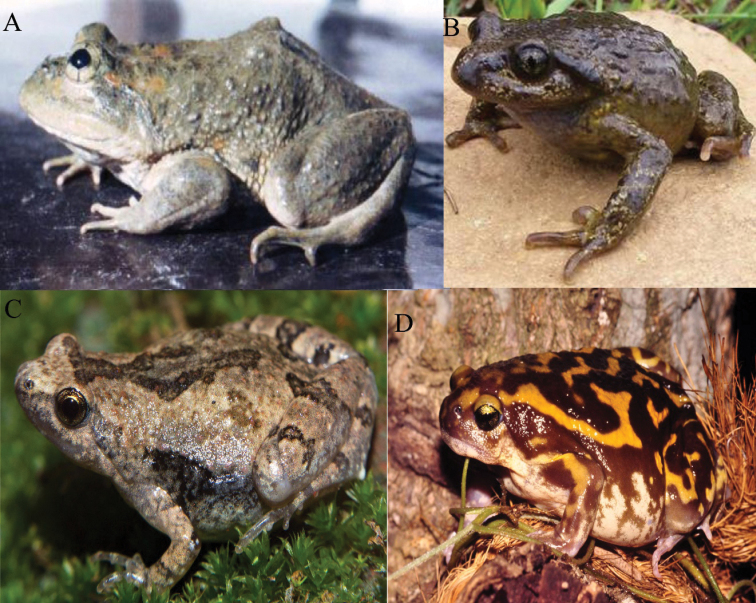
**A** Karez Frog (*Chrysopaasternosignata*) **B** Ladakh Pelobatid Toad (*Scutigeroccidentalis*) **C** Ant Frog (*Microhylanilphamariensis*) **D** Marbled Balloon Frog (*Uperodonsystoma*). Photographers: Dr Muhammad Rais (**A**); Dr Matthias Stöck (**B**); Chaitanya Shukla (**C**); Peter Janzen (**D**).

**Dicroglossidae**

**Table:** 

1	Tympanum indistinct, body dorsum brownish, smooth with a few tubercles on flanks, dark bars on forearm, thighs and shank	***Nanoranavicina* (Stoliczka, 1872) (Fig. 3D)**
–	Tympanum distinct	**2**
2	Toes partially webbed, snout pointed	***Minervaryapierrei* (Dubois, 1975) (Fig. 3C)**
–	Toes completely webbed	**3**
3	Inner metatarsal tubercle shovel-shaped	***Sphaerothecamaskeyi* (Schleich and Anders 1998) (Fig. 3 E**–**F)**
–	Inner metatarsal tubercle elongate	**4**
4	Body dorsum with longitudinal folds and mid-dorsal line	***Hoplobatrachustigerinus* (Daudin, 1802) (Fig. 2F)**
–	Body dorsum without longitudinal folds	**5**
5	Body pustules large, multispinulate, belly spiny	***Chrysopaasternosignata* (Murray, 1885) (Fig. 4A)**
–	Body pustules small, unispinulate, belly spineless	**6**
6	Nuptial spines absent	**7**
–	Nuptial spines present	**8**
7	Ventral body spotted, relative length of fingers 4<2<1<3	***Euphlyctiscyanophlyctis* (Schneider, 1799) (Fig. 3A)**
–	Ventral body whitish, relative length of fingers 1=2 < 4< 3	***Euphlyctiskalasgramensis* (Howlader et al., 2015**b) **(Fig. 3B)**
8	Spinules on pustules	***Allopaabarmoachensis* (Khan and Tasnim, 1989) (Fig. 2D)**
–	Spinules on longitudinal ridges	***Allopaahazarensis* (Dubois and Khan, 1979) (Fig. 2E)**

### Statistical comparisons of *Sphaerotheca* populations

Of the studied 23 morphometric measurements in *S.maskeyi*, we obtained from our PCA 10 and eight significant variables (*r* > 0.90) in males having uniform rusty-colored dorsum and having dotted pattern, respectively. We obtained 10 and one significant variables (*r* > 0.90) in females, respectively. Eigen value, variability (%), cumulative variability (%), and factor loadings of the 23 morphometric measurements are given Table 1.

The multivariate generalized linear model revealed statistically significant difference (*F*_(1, 11)_ = 1876.60, *P* = 0.018; Wilk’s Λ = 0.00, partial η^2^ = 0.97) in the morphometric measurements of males but non-significant in the females (*F*_(13, 11)_ = 0.944, *P* = 0.532; Wilk’s Λ = 0.556, partial η^2^ = 0.444) of the two forms of *S.maskeyi*.

**Table 1.** Eigen value, variability (%), cumulative variability (%) and factor loadings of the 23 morphometric measurements of the two forms (uniform rusty-colored dorsum and dorsum olive with dotted pattern) of *Sphaerothecamaskeyi*. The factor loadings with absolute correlation values greater than 0.90 were considered significant (in bold).

**Male**		**Uniform rusty-colored dorsum**			**Dorsum olive with dotted pattern**		
		**F1**	**F2**	**F3**	**F1**	**F2**	**F3**
	**Eigenvalue**	10.112	7.146	5.742	14.476	2.872	1.934
	**Variability (%)**	43.964	31.069	24.966	62.940	12.486	8.409
	**Cumulative** %	43.964	75.034	100.000	62.940	75.425	83.835
	**Factor loadings**						
	**Morphometric measurements**	**F1**	**F2**	**F3**	**F1**	**F2**	**F3**
	Snout–vent length	0.273	−0.113	**0.955**	0.864	0.383	−0.170
	Head width	−0.195	**0.936**	0.293	**0.977**	0.066	−0.142
	Head length	−0.224	0.845	0.486	**0.984**	0.014	−0.058
	Distance between nostrils	**0.970**	−0.213	−0.114	0.736	0.333	0.383
	Width of upper eyelid	0.613	0.232	−0.755	0.764	−0.522	0.237
	Interorbital distance	0.868	0.056	−0.493	0.268	−0.174	**0.916**
	Distance from the back of the mandible to the nostril	0.003	**0.919**	0.393	0.754	0.521	−0.286
	Distance from the back of the mandible to the front of the eye	0.070	0.856	−0.513	0.826	−0.300	−0.059
	Distance from the back of the mandible to the back of the eye	−0.488	0.677	−0.552	0.853	−0.465	−0.174
	Distance between the front of the eyes	0.811	−0.580	0.072	0.770	0.263	−0.360
	Distance between back of the eyes	**0.964**	0.005	−0.266	0.286	0.603	0.624
	Distance from the front of the eye to the nostril	**0.901**	0.430	−0.054	0.827	0.106	0.030
	Eye length	−0.924	0.384	0.006	**0.953**	−0.165	−0.101
	Distance from the nostril to the tip of the snout	−0.846	−0.158	−0.509	**0.902**	−0.365	−0.201
	Distance from the front of the eye to the tip of the snout	**0.941**	−0.218	−0.261	**0.950**	0.084	0.064
	Greatest tympanum diameter	−0.660	−0.633	−0.404	**0.915**	−0.136	0.165
	Distance from tympanum to the back of the eye	−0.107	0.468	−0.877	0.807	−0.201	0.176
	Forelimb length	−0.641	−0.763	−0.086	0.874	−0.257	−0.002
	Hand length	**0.987**	−0.005	0.160	0.745	0.170	0.065
	Femur length	0.639	0.627	−0.446	0.829	−0.006	−0.187
	Shank length	−0.267	**0.959**	−0.095	0.783	0.088	0.216
	Length of tarsus and foot	0.685	0.293	0.666	0.086	**0.926**	−0.074
	Foot length	−0.033	0.252	**0.967**	0.731	0.313	−0.012
**Female**		**Uniform rusty−colored dorsum**			**Dorsum olive with dotted pattern**		
		**F1**	**F2**	**F3**	**F1**	**F2**	**F3**
	**Eigenvalue**	12.044	5.877	4.595	12.166	4.445	2.062
	**Variability (%)**	52.364	25.552	19.979	52.895	19.325	8.967
	**Cumulative** %	52.364	77.916	97.895	52.895	72.221	81.188
	**Factor loadings**						
	**Morphometric measurements**	**F1**	**F2**	**F3**	**F1**	**F2**	**F3**
	Snout–vent length	0.804	0.364	0.471	0.245	0.144	−0.476
	Head width	**0.973**	0.229	0.006	0.895	0.154	0.078
	Head length	0.785	−0.164	−0.597	0.774	0.451	0.368
	Distance between nostrils	0.584	0.791	0.171	0.827	0.029	−0.143
	Width of upper eyelid	0.576	−0.402	0.709	0.833	−0.384	−0.190
	Interorbital distance	−0.244	0.127	**0.961**	0.603	0.095	−0.648
	Distance from the back of the mandible to the nostril	0.790	0.320	−0.476	0.659	0.097	0.673
	Distance from the back of the mandible to the front of the eye	0.766	−0.614	−0.190	0.786	0.160	0.412
	Distance from the back of the mandible to the back of the eye	0.860	−0.509	−0.030	0.881	−0.332	0.194
	Distance between the front of the eyes	0.124	**0.987**	−0.072	0.401	0.807	−0.124
	Distance between back of the eyes	−0.919	0.285	0.272	−0.045	0.897	−0.309
	Distance from the front of the eye to the nostril	0.381	**0.925**	−0.014	0.546	0.575	0.014
	Eye length	**0.999**	−0.034	−0.015	0.826	−0.479	0.196
	Distance from the nostril to the tip of the snout	**0.992**	−0.083	0.094	0.879	−0.333	−0.108
	Distance from the front of the eye to the tip of the snout	0.188	0.488	0.842	0.743	0.370	−0.044
	Greatest tympanum diameter	**0.993**	0.066	0.093	0.870	−0.436	−0.032
	Distance from tympanum to the back of the eye	0.845	−0.533	0.035	0.858	−0.295	−0.274
	Forelimb length	**0.910**	−0.288	0.297	0.877	−0.431	0.028
	Hand length	0.435	0.865	0.221	0.860	0.217	−0.108
	Femur length	0.219	−0.156	**0.961**	0.731	0.154	−0.382
	Shank length	**0.964**	−0.263	−0.011	0.804	0.277	−0.130
	Length of tarsus and foot	0.051	0.606	−0.501	−0.191	**0.908**	0.159
	Foot length	0.657	0.623	−0.422	0.672	0.442	0.344

## Discussion

A number of researchers have documented the amphibian fauna of Pakistan; Pratihar et al. (2014) reported 25 species, Khan (2014) 24 species, Sarwar et al. (2016) 21 species, and Ali et al. (2018) 26 species, but these studies did not arise from any systematic survey of the country or regions of the country, nor did they employ a molecular taxonomic approach. To date, much of the difficult terrain, especially in the high-altitude northern and arid western mountains of the country, has remained unexplored.

The true toads of Pakistan are represented by two genera: *Duttaphrynus*Frost et al., 2006 and *Bufotes* Rafinesque, 1815. *Duttaphrynus* is characterized by prominent ridges on the head, while *Bufotes* lacks such ridges but bears conspicuous pattern of irregularly shaped, darker, green or greenish-olive spots.

Considering other taxa, Faiz et al. (2018) reported three amphibians, including *Allopaabarmoachensis* from Toli Pir National Park, Pakistan. Dubois (1992) considered *A.barmoachensis* synonymous with *Allopaahazarensis*, but Khan (2004) regarded the two as distinct. However, Ohler and Dubois (2006) reiterated that the species is conspecific with *A.hazarensis*. As no molecular data exist to separate the two species, there is no evidence for separation. The species complex of *Euphlyctis* also needs detailed study. Dutta (1997) provided a record of *Euphlyctishexadactylus* from Pakistan which needs confirmation. Murray (1884) reported and described *Tomopternastrachani* from Sindh, Pakistan, and Khan (2006) reported the species as *Sphaerothecabreviceps*. Dubois (1999) suggested that *S.breviceps* is a small-sized species based on the study of the name-bearing specimens. For the large-sized species of South Asia, he suggested that the names *Ranavariegata* (Gravenhorst, 1829); *Pyxicephalusfodiens* (Jerdon, 1853); *Pyxicephaluspluvialis* (Jerdon, 1853); *Sphaerothecastrigata* (Günther, 1859); *Ranadobsonii* (Boulenger, 1882); *Tomopternastrachani* (Murray, 1884); *Ranaleuchorhynchus* (Rao, 1937); and *Ranaswani* (Myers and Leviton, 1956) are available. Dubois (1999) also regarded *Tomopternamaskeyi* to be a provisional synonym of these large-size taxa. Recently, Deepak et al. (2020) provided distribution records for *Sphaerothecapashchima* from India and morphological descriptions of their samples of *S.pashchima* match samples collected for the present study. Deepak et al. (2020) reported similar morphological variation in *S.pashchima* among the samples collected from India. Khatiwada et al. (2021) demonstrated high similarity between topotypical material of *S.maskeyi* with name-bearing types of *S.pashchima* and considered the later name a synonym of *T.maskeyi*, valid as *S.maskeyi*. Molecular identification of our samples also confirms their identity as *S.maskeyi* (see Akram et al. 2021). Therefore, we conclude that *Sphaerothecamaskeyi* occurs in Pakistan, and not *Sphaerothecabreviceps* as reported by Murray (1884) and Khan (2006).

Borthakur et al. (2007) studied cricket frog species in Assam, northwest India (*Fejervaryanepalensis*, *F.pierrei* Dubois, 1975, *F.syhadrensis* Annandale, 1919, and *F.teraiensis* Dubois, 1984), which have been assigned to other genera in Nepal by Ahmed et al. (2009) and Shah and Tiwari (2004). Rawat et al. (2020) reported *Minervarya* species from extreme southwestern Nepal in the Shuklaphanta National Park. Molecular identification of our samples confirms their identity as *Minervaryapierrei* (see Akram et al. 2021). Two distinct forms of *Minervarya* are known, one with a mid-dorsal stripe and another without it. Dubois (1974) has reported such variation. Hence, we suggest conducting country-wide surveys and use a molecular approach to confirm presence of other species of *Euphlyctis*, *Sphaerotheca*, and *Minervarya* from Pakistan.

The inclusion of *Uperodonsystoma* in the list of amphibians of Pakistan is based on two reports. Baig and Gvozdik (1998) reported this species from a torrent stream in the Shakarparian Hills, Islamabad Capital Territory (ICT), and Masroor (2011) recorded this species from a subtropical, semi-evergreen forest in Margalla Hills National Park (ICT). We consider *U.systoma* to be very rare in Pakistan. No historical quantitative data has been found to date. Some species assessed as Least Concern by the IUCN, such as *U.systoma*, are considered rare in Pakistan, compared to elsewhere in their global range. Hence, we caution the use of global conservation status for the amphibian species that occur within Pakistan.

### Future prospects in amphibian research and conservation in Pakistan

Pakistan represents the westernmost limit of the geographic range of *Duttaphrynusmelanostictus*. This species has been introduced outside its natural range into many parts of the world, and in these places it is considered a nuisance predator, a potential disease vector, and the cause of many other ecological problems (Labisko et al. 2015; Piludu et al. 2015). Studying the ecology and biology of *D.melanostictus* in its native range could help manage this species in Pakistan as well as elsewhere.

The chytrid fungus *Batrachochytriumdendrobatidis* affects amphibians worldwide. The likelihood of this fungus occurring in Pakistan is predicted to be low (<30%) (Olson et al. 2013; Rodder et al. 2010) by models which did not include samples of anurans from Pakistan. This lack of data may produce inaccurate results in models, which use no direct observational data. Therefore, the study of the prevalence of chytrid fungus in countries such as Pakistan is important to fill in these data gaps. Furthermore, the northern regions of Pakistan have complex and dynamic ecosystems (Roberts 1997) and therefore more diverse amphibian assemblages. Diversity of amphibians in an ecosystem has been linked to increased probability of the introduction and spread of chytrid fungus (Olson et al. 2013). This correlation with amphibian diversity and the lack of data in the Middle East and South Asia creates an urgency to perform risk assessments on amphibian communities in these regions.

There is also a dire need to change social attitudes towards amphibians in our society. This could be achieved by initiating community awareness by outreach, school, and citizen-science programs. While designing research projects, special attention should be given to include components of outreach. For instance, people working in agroecosystems can organize field activities with farmers and local communities. Likewise, the ongoing 10 Billion Tree Tsunami project by the Ministry of Climate Change, Government of Pakistan, should integrate consideration for herpetofauna species, particularly anuran species such as *Allopaahazarensis* and *Allopaabarmoachensis*, which are endemic to forested montane wetlands. The development of android applications and websites could help reach out to the public. This, however, would be limited to those people who have access to the internet, but their participation would inevitably enhance the documentation of species occurrence and distribution records in the country. Collection and archiving quantitative data on anuran abundance would also help determine the current conservation status of our anuran species.

We suggest setting research priorities and to devise strategy for the conservation of amphibians of Pakistan when manageable anthropogenic threats exist, such as habitat destruction, urbanization, pollution, and unsustainable utilization, so that amphibian populations can be better controlled by utilizing less financial, administrative, and human resources. This can be achieved through short-, medium-, and long-term actions. Short-term actions could include the establishment of a network or people currently engaged in amphibian related research. A conservation assessment and management plan workshop should be organized wherein experts and researchers could provide their opinions and draft recommendations for medium- and long-term actions.

A medium-term action plan may include carrying out systematic and coordinated surveys throughout the country to establish a database on occurrence and distribution of species and the identification of their threats. It is recommended to use modern taxonomic tools, such as DNA barcoding, to determine taxonomy and initiate research on phylogenetic affinities, biogeography systematics, especially on endemic species. This approach can expect to yield additional amphibian species as a result. Some genera, such as *Microhyla* Tschudi, 1838, *Uperodon* Duméril & Bibron, 1841, *Minervarya*Dubois et al., 2001), *Allopaa*, *Chrysopaa* Ohler & Dubois, 2006, *Euphlyctis* Fitzinger, 1843, *Nanorana*, and *Sphaerotheca* Günther, 1896, which occur in Pakistan need additonal data for molecular taxonomy and detailed comparisons with taxa in other South Asian countries.

Long-term actions would entail monitoring of amphibian populations, threat mitigation, and appropriate legislation. Amphibians have been excluded from all current legislative and policy decisions of the country. The National Climate Change Policy (GoP 2012), the Pakistan National Biodiversity Strategy and Action Plan (GoP 2015), the Biodiversity Action Plan of Pakistan (GoP 2000), and the Pakistan National Conservation Strategy (GoP 1992) do not currently support the need to carry out research and conserve amphibians. Likewise, amphibians are not protected under any law (Shafiq 2005). Hence, the legislation pertaining to threatened and endemic species needs to be updated, particularly in need of revision is Schedule III, which includes protected species, of provincial and federal wildlife laws as well as the CITES appendices.

Wildlife conservation projects in Pakistan mainly focus on carnivores, ungulates, and birds. Shehzad et al. (2012) reported the occurrence of *Nanoranavicina* in the diet of *Prionailurusbengalensis* in Ayobia National Park, Khyber Pakhtunkhwa, Pakistan. Such studies usually lack the mandate of investigating whether a particular food item was eaten directly or through an alternate dietary item. Whatever the case, this explicitly signifies the role of amphibians in the food chain and could be used as an impetus to incorporate amphibians in such research projects and conservation programs. Therefore, it should be proposed to adopt an inclusive wildlife conservation approach in Pakistan. The approach would advocate the integration of poorly documented taxa, such as amphibians, in wildlife conservation and management projects to enhance the significance of their existence and the intrinsic values of all wildlife species which would eventually ensure their continued survival.

## Availability of data

The data underpinning the analysis reported in this paper are deposited in the Dryad Data Repository at Dryad (https://doi.org/10.5061/dryad.mkkwh7118).

## Supplementary materials

Supplementary material 1 Supplementary tables Data type statistical data Explanation note **Table S1.** Sample ID and Snout-vent length of samples examined. **Table S2a.** Descriptive statistics of morphometric measurements of *Sphaerothecamaskeyi* (male). **Table S2b.** Descriptive statistics of morphometric measurements of Sphaerothecamaskeyi (female). https://binary.pensoft.net/file/599600This dataset is made available under the Open Database License (http://opendatacommons.org/licenses/odbl/1.0/). The Open Database License (ODbL) is a license agreement intended to allow users to freely share, modify, and use this Dataset while maintaining this same freedom for others, provided that the original source and author(s) are credited.Muhammad Rais, Waseem Ahmed, Anum Sajjad, Ayesha Akram, Muhammad Saeed, Hannan Nasib Hamid, Aamina Abid
